# Psychometric properties and measurement invariance of the Arabic Self-Care Inventory

**DOI:** 10.1371/journal.pone.0291904

**Published:** 2023-09-20

**Authors:** Jehad A. Rababah, Mohammed Munther Al-Hammouri, Michela Luciani

**Affiliations:** 1 Faculty of Nursing, Jordan University of Science and Technology, Irbid, Jordan; 2 Postdoctoral Fellow in Nursing, Department of Medicine and Surgery, University of Milano-Bicocca, Milano, Italy; The World Islamic Sciences and Education University, JORDAN

## Abstract

**Background:**

Self-care is a fundamental aspect of health and well-being for healthy individuals and those with chronic illnesses. However, the available self-care measurement instruments have limited support regarding their psychometric properties. Research about the validation of comprehensive, theory-based self-care tools in the Arabic language and culture is also limited. In addition, many self-care measurement tools are available only for people with chronic illnesses.

**Objective:**

To examine the psychometric properties of the Arabic version of the Self-care Inventory (SCI) in the general adult population in Jordan.

**Methods:**

This study was conducted using a cross-sectional design. Data collection was performed using a demographics questionnaire, and Arabic versions of the SCI, Self-care Self-Efficacy, and the Center for Epidemiologic Studies Depression Scale-revised. SPSS and AMOS were used to analyze the data. Data analysis was conducted by performing confirmatory factor analysis, measurement invariance, internal consistency, and bivariate correlations.

**Results:**

The results revealed that the SCI comprises three scales: self-care maintenance, self-care monitoring, and self-care management. The goodness of mode fit indices showed that the models of these scales fit the data well by meeting the following set a priori criteria: (RMSEA < .07, CFI > .95, and X^2^/df < 5). The factor loadings of the individual items of the SCI provided further evidence about the factor structure of the three scales. Regarding measurement invariance, the results indicated that partial invariance across participants’ sex is assumed. The values of both Cronbach’s α and composite reliability showed that the internal consistency of the SCI is supported. Cronbach’s α of the self-care maintenance, self-care monitoring, and self-care management were .82, .86, and .83, respectively.

**Conclusion:**

The psychometric properties of the Arabic version of the SCI demonstrate its validity and reliability as a robust assessment tool for measuring self-care in the general adult population.

## Introduction

Self-care is a fundamental aspect of health and well-being for healthy individuals and those with chronic illnesses. The Middle-Range Theory of Self-Care of Chronic Illness defines self-care as "a process of maintaining health through health-promoting practices and managing illness" [1 p 208]. Self-care is considered as a decision-making process demonstrating the individual’s knowledge about their overall health as well as the capabilities for engaging in health promoting activities [2]. According to Riegel and colleagues [1], the three main concepts of self-care are: 1) self-care maintenance, 2) self-care monitoring, and 3) self-care management. These three concepts comprehensively cover the essential aspects of maintaining health and managing symptoms upon occurrence. To achieve high levels of self-care, these three concepts should be integrated instead of implementing them separately.

Self-care has been described in the literature as the actions individuals take to promote and maintain their physical, psychological, mental, social, spiritual well-being [3, 4]. Individuals involved in self-care activities are often capable of achieving holistic care and health promotion. A variety of self-care strategies, including physical activity and relaxation techniques, can help individuals manage stress and maintain a sense of balance and well-being. Despite the importance of self-care, many people do not engage in adequate self-care behaviors, which can lead to negative consequences for their health and well-being [5–7]. A number of factors, including time constraints, lack of motivation, competing demands, financial constraints, lack of knowledge, and negative attitudes towards self-care, can discourage individuals from prioritizing self-care [5, 6, 8, 9].

In recent years, a number of self-care tools have been developed to help individuals assess and improve their self-care practices. However, certain limitations of many of these tools should be addressed. Two recent systematic reviews showed that the available self-care measurement instruments have limited support regarding their psychometric properties [10, 11]. Furthermore, many self-care measurement tools are available for people with chronic illnesses like heart failure and diabetes mellitus. While it is necessary for researchers to consider the characteristics of the study population and the setting before selecting the self-care instrument, most tools that are designed for the general adult population are not theory-based [12]. To address these issues, the Self-care Inventory (SCI) has been developed. The SCI is grounded in the Middle-Range Theory of Self-Care of Chronic Illness and specifically tailored to measure self-care in the general adult population [12]. More information about the SCI is provided in the methods section.

From a cultural standpoint, most self-care measurement tools have been developed in Western cultures and may not be fully applicable or relevant to individuals from other cultural backgrounds [13]. The Arabic language and culture are important contexts to consider when developing self-care tools, as Arabic-speaking individuals may have different self-care needs and practices compared to those from Western cultures [14]. However, there is a lack of research on the development and validation of comprehensive, theory-based self-care tools in the Arabic language and culture. Thus, in this study the authors aim to examine the psychometric properties of the Arabic version of the SCI in the general adult population in Jordan. This involves evaluating the internal consistency, factor structure, measurement invariance, and convergent and divergent validity. By examining the validity and reliability of this tool, the authors hope to contribute to the development of a culturally relevant self-care resource that can be used among Arabic-speaking individuals.

Examining the psychometric properties of the SCI necessitates examining the association between self-care and other concepts. Based on the available evidence from the literature, the authors selected self-care self-efficacy, and depressive symptoms to evaluate SCI convergent and divergent validity. Self-care self-efficacy is considered a distinct factor that influences self-care [15]. Self-care self-efficacy is defined as " the confidence that one has in the ability to perform a specific action and to persist in performing that action despite barriers" [15 p 15]. On the other hand, poor self-care is associated with higher risk for experiencing depressive symptoms [16–18]. Therefore, the authors expect that the different dimensions of self-care will demonstrate a positive correlation with self-care self-efficacy and a negative correlation with depressive symptoms.

## Methods

### Design and setting

This study was conducted using a cross-sectional design in a sample of Jordanian adults.

### Participants

The SCI is intended to be used in the general adult population regardless of any underlying health conditions or medical history [12]. Therefore, the only inclusion criterion to participate in this study was age; ≥ 18 years old. Convenience sampling approach was applied in this study. Invitations to participate in this study were posted on social media and in public settings. Regarding the sample size, Kline maintained that recruiting 10 to 20 per model parameter (i.e., item of the SCI) is sufficient to conduct factor analysis [19]. Because the SCI is a 20-item tool, the adequate sample size to conduct this study was initially estimated between 200 and 400 Jordanian adults. However, the final sample size was determined by taking into account that measurement invariance, which is considered a multiple group analysis, would be performed and necessitated larger sample sizes. Measurement invariance was performed to obtain in-depth insights about the construct validity of the SCI. In addition, measurement invariance provides additional evidence regarding the validity of the scale across sex and minimizes the likelihood of measurement bias [19, 20]. Therefore, the authors intended to recruit more than 400 Jordanian adults to conduct measurement invariance analysis based on the sex of the participants. A total of 521 adults were recruited (238 males and 283 females) which is considered a sufficient sample to address the study objectives. The recruitment of participants and data collection in this study started on April 25^th^, 2023, and ended on June 3^rd^, 2023. The estimated response rate in the current study was approximately 60%.

### Data collection

A link to complete the data collection scales was sent to the participants who agreed to participate in this study. The link was sent via emails and mobile phone messages to ensure convenience access and secure responses. The data collection scales included a demographics questionnaire, and Arabic versions of the SCI, Self-care Self-Efficacy (SCSE), and the Center for Epidemiologic Studies Depression Scale-revised (CESD-R). The demographics questionnaire included questions about participants’ age, sex, marital status, and education. Google Forms were used to create the data collection instruments. The first part of the Google Forms presented the participant with information about the study, including its purpose, methods, risks, benefits, and confidentiality measures. The last sentence of this section asked whether the participant agreed or declined to participate in the study. No identifying data were collected from the study participants.

#### Self-care

The SCI was used to measure self-care [12]. This tool consists of 20 items listed under three separate scales: 1) self-care maintenance (8 items), 2) self-care monitoring (6 items), and 3) self-care management (6 items). The response options for the items are Likert type with 5 possible options. The total score is obtained using specific instructions and formulas available on the web [21]. The resulting total scores for the three scales are out of 100; scores of ≥70 indicate adequate self-care. The English version of this tool have been validated in a sample of US citizens and the evidence supports its reliability and validity [12].

#### Self-care self-efficacy

The developers of the SCI recommend using another scale to measure self-care self-efficacy. Self-care Self-Efficacy (SCSE) Scale is a 10-item tool used to measure the individual’s confidence to perform and maintain self-care [21]. The response options for the SCSE scale are Likert type with possible scores for each item ranging from 1 (Not Confident) to 5 (Extremely Confident). Obtaining total SCSE scale scores are also available on the web [21]. The psychometric properties of this scale have been examined in samples recruited from the USA, Hong Kong, Italy, and Brazil [22].

#### Depressive symptoms

The Center for Epidemiologic Studies Depression Scale-Revised (CESD-R) was used to measure depressive symptoms [23]. CESD-R is a 20-item scale with 5-point Likert-like answer options from 0 (not at all or less than 1 day) to 4 (nearly every day for 2 weeks) [23]. The possible total score ranges from zero to 80, and a score equal to or above 16 indicates a person is at risk for clinical depression [23]. The Arabic version of this tool has been validated in samples of young adults and the results supported the psychometric properties and factor structure of the scale [24].

### Translation process

The SCI and SCSE scales were translated into Arabic following the process and terms of translating self-care instruments [21]. The principal investigator obtained permission to translate the instruments from Dr. Michela Luciani, the developer of the original scales. The original scales (available in English) were first translated by two certified translators into Arabic. The researchers then met with the translators to finalize the translated version and develop the final versions of the forward translated scales. After that, two independent certified translators back translated the Arabic versions into English. The researchers also met with those translators to finalize the back translated versions of the scales. The final versions (both Arabic and English) were reviewed by the authors of the original scale and revised according to their comments. The final approved Arabic versions are available on self-care-measures.com.

### Data analysis

Descriptive statistics, using SPSS (version 25), were used to summarize the data and to describe the characteristics of the sample. SPSS was used to obtain Cronbach’s alpha for the scales used in the current study to examine the internal consistency of the SCI scales. Composite reliability was also examined and values of ≥ .70 were considered supportive of factor convergence [20]. In addition, SPSS was used to examine the relationship between self-care and other variables of interest (i.e., self-care self-efficacy, and depressive symptoms). It is worth noting that exploratory factor analysis using SPSS was not performed in this study. That is because the original SCI is a theory-grounded tool with well-established items and factor structure [12].

The authors performed confirmatory factor analysis (CFA), using AMOS (version 25), to examine the factor structure of the SCI. Different models were created based on the available theoretical and empirical evidence regarding the factor structure of the SCI [12, 25]. To compare these models and assess the fit of the model, the author used a number of goodness-of-fit measures, such as the chi-square statistic, chi-square/degrees of freedom, root mean square error of approximation (RMSEA), and comparative fit index (CFI). An acceptable fit of the model was indicated by a non-significant chi-square statistic, chi-square/degrees of freedom (X^2^/df) of less than 5, an RMSEA of less than 0.07, and a CFI of greater than 0.95 [19, 20]. In addition, factor loadings were evaluated with .50 and .70 values were considered acceptable and ideal, respectively. Using AMOS, the authors also examined three different types of measurement invariance (configural, metric, and scalar invariance) of the SCI [19, 20, 26]. Item #14 (Think about the last time you had a symptom. This can be a symptom of anything–a cold, a bad night sleep, an illness. It could also be a reaction to a medicine) of the SCI was not entered into the model of self-care monitoring in the factor analysis following the recommendation of Luciani and colleagues to consider this item as a measure of symptom recognition [12].

### Ethical considerations

The current research was approved by the institutional review board (IRB) committee at Jordan University of Science and Technology (ID: 43/153/2023). All participants provided electronic informed consent after reading the information about the study, including its purpose, methods, risks, benefits, and confidentiality measures.

## Results

### Participants characteristics

The average age of the participants in this study was 32.56 (*SD* = 8.19). More than half of the participants in this study were female (54.3%) and married (52.8%). The average scores on the self-care maintenance, self-care monitoring, and self-care management were 52.09 (*SD* = 20.63), 54.01 (*SD* = 23.02), and 49.23 (*SD* = 22.49); respectively. Table 1 provides a summary of the participants’ characteristics and the average scores of the data collection tools.

**Table 1:** Participants’ demographic characteristics (N = 521).

Variable	Frequency	Percentage
Sex Male Female	238 283	45.7% 54.3%
Marital status Single Married Divorced Widow(er)	197 275 42 7	37.8% 52.8% 8.1% 1.3%
Highest level of education ≤ High school Diploma Bachelor’s Graduate	92 45 364 21	17.7% 8.6% 69.9% 3.8%
Smoking status No Yes	422 99	81% 19%
	Mean (SD)	Minimum-Maximum
Age (years)	32.56 (8.19)	18–65
Self-care maintenance	52.09 (20.63)	0–100
Self-care monitoring	54.01 (23.02)	0–100
Self-care management	49.23 (22.49)	0–100
Self-care self-efficacy	56.27 (22.41)	0–100
CESD-R	24.13 (16.63)	0–80

SD: standard deviation; CESD-R: the Center for Epidemiologic Studies Depression Scale-revised.

### Confirmatory factor analysis

Different versions of the SCI scales were evaluated using AMOS. Table 2 presents the values of the goodness of model fit indices for the different versions of the scales. As presented, the two-factor version of the self-care maintenance scale had better indices of goodness of model fit (X^2^ {18} = 55.75, p < .001, RMSEA = .064, CFI = .964, X^2^/df = 3.10) compared to the single factor version of the scale. The single factor version of the self-care monitoring scale demonstrated excellent fit to the data (X^2^ {9} = 16.53, p = .06, RMSEA = .040, CFI = .994, X^2^/df = 1.84). Therefore, no other versions of self-care monitoring were examined. Compared to the single factor version of the self-care management scale, the two- factor version had better goodness of model fit indices (X^2^ {8} = 27.74, p = .001, RMSEA = .069, CFI = .981, X^2^/df = 3.47).

**Table 2:** Goodness of model fit indices of the different versions of the SCI scales.

Scale & Model	*X* ^ *2* ^ , *df (sig*.*)*	RMSEA	CFI	X^2^/df
**Self-care maintenance:**				
Single factor	75.30, 20 (< .001)	.073	.948	3.77
Two factors	55.75, 18 (< .001)	.064	.964	3.10
**Self-care monitoring:**				
Single factor	11.49, 5 (.04)	.050	.994	2.30
**Self-care management:**				
Single factor	95.64, 9 (< .001)	.136	.917	10.63
Two factors	27.74, 8, (.001)	.069	.981	3.47

X^2^: chi-square statistic; df: degrees of freedom; sig: p value; RMSEA: root mean square error of approximation; CFI: comparative fit index; X^2^/df: chi-square/degrees of freedom.

**Fig 1:**
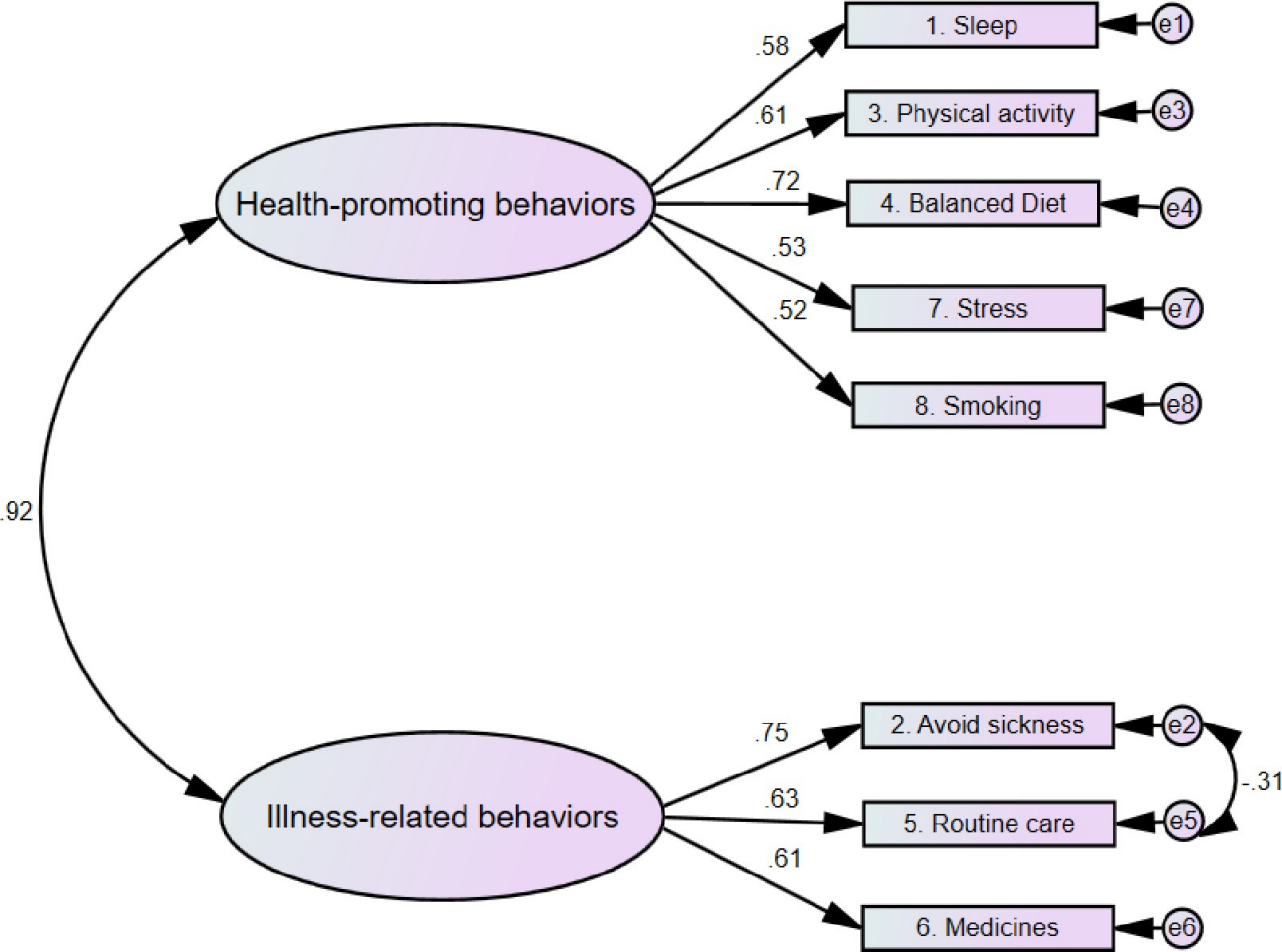
Self-care maintenance scale.

**Fig 2:**
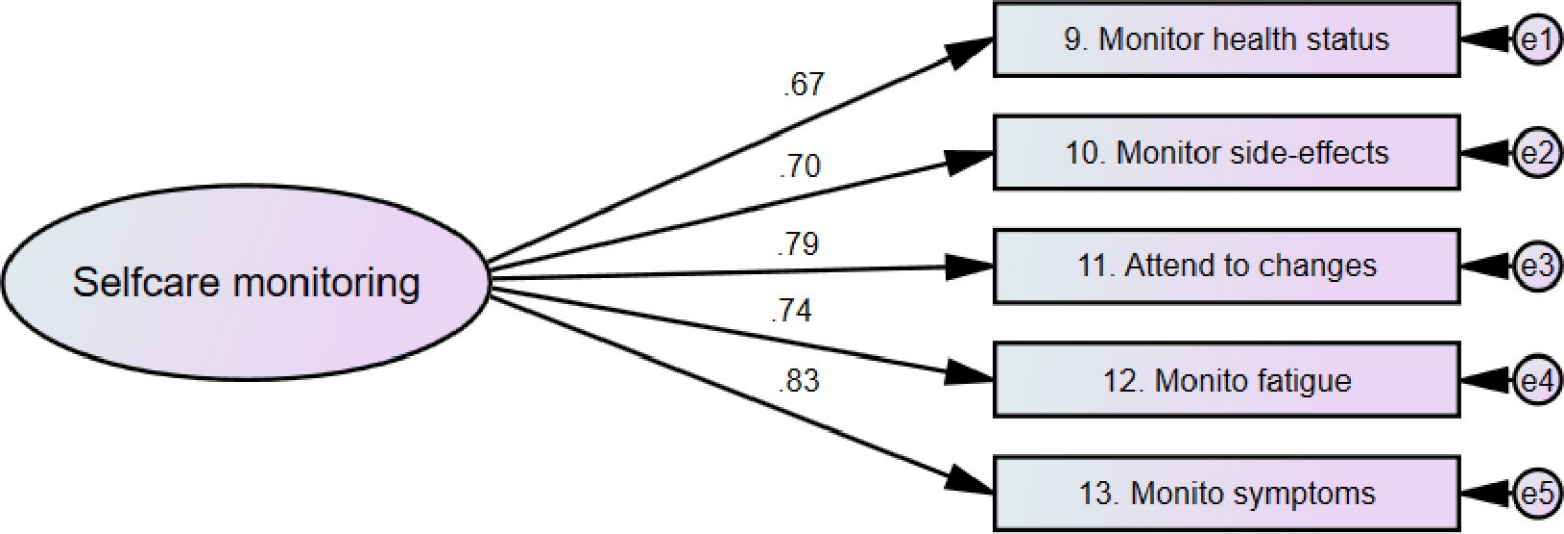
Self-care monitoring scale.

**Fig 3:**
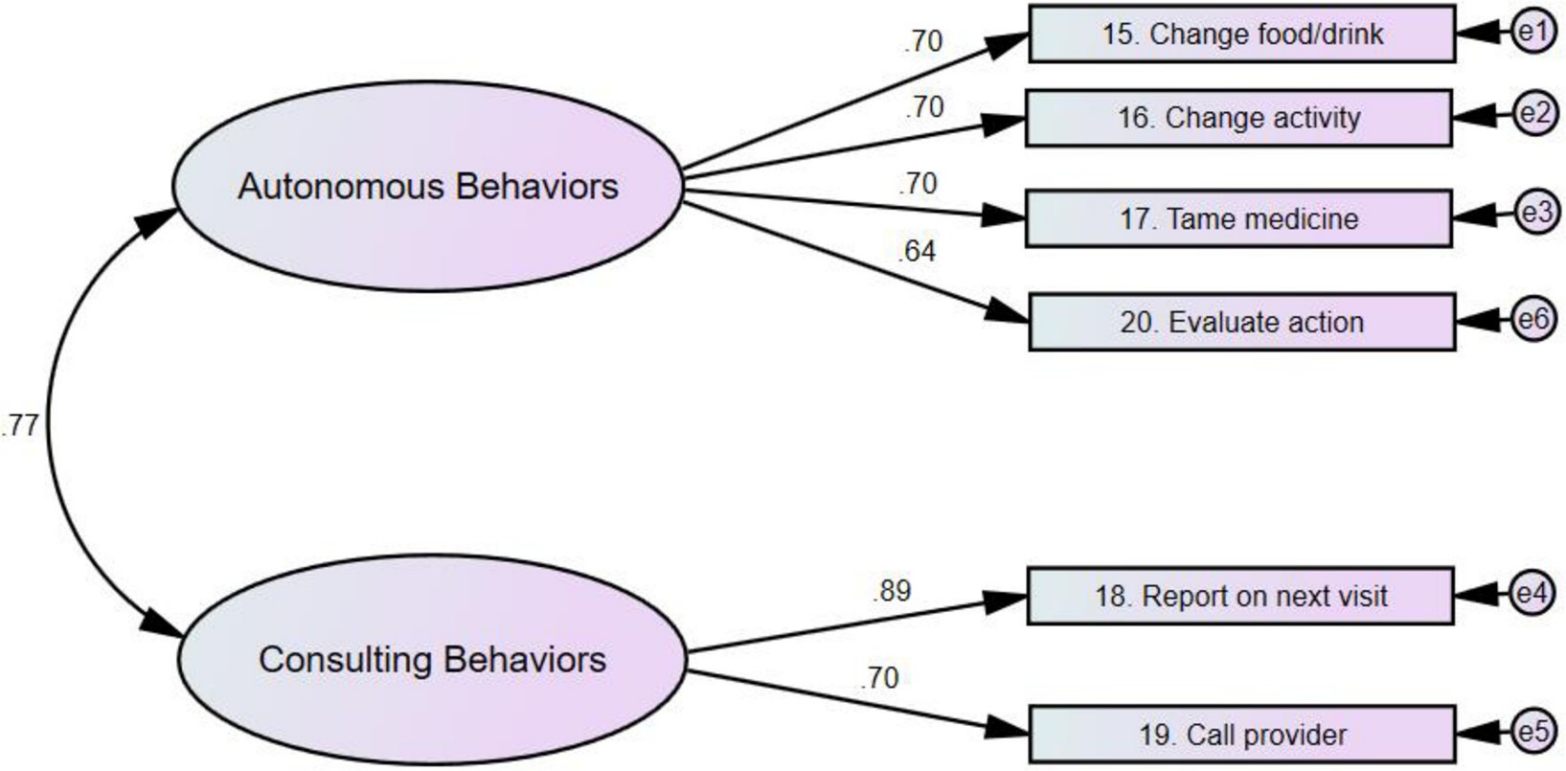
Self-care management scale.

Another examined parameter during CFA was the factor loadings of the individual items of the SCI. The outputs of AMOS analyses revealed that 11 of the SCI items had ideal factor loadings, whereas the remaining nine items had good factor loadings. The two factors of self-care maintenance were health-promoting and illness-related behaviors. Two items of the self-care maintenance had ideal factor loadings (>.70) while the remaining six items were good (>.50). Self-care monitoring scale had a single factor with five items; with factor loadings of four of these items were ideal. The self-care management scale had two factors (autonomous and consulting behaviors) and the factor loadings of five out of the six items were ideal. The final models of the SCI scales along with the factor loadings of the items are presented in Figs 1–3.

### Simultaneous confirmatory factor analysis of the SCI

The authors intended to examine whether the three scales of SCI and their pertinent factors reflect the construct self-care. Therefore, all items of the SCI were analyzed simultaneously in AMOS. The goodness of model fit indices, factor loadings, and composite reliability coefficients were all evaluated in this step. The fit indices showed that the entire model was acceptable: X^2^ (141) = 378.18, p < .001, RMSEA = .057, CFI = .942, X^2^/df = 2.68. In this simultaneous model, the factor loadings of eight items of the SCI were ideal whereas the remaining items demonstrated acceptable values. Regarding composite reliability, all factors had values of ≥.70 except illness-related behaviors (Table 3).

**Table 3:** Results of the simultaneous confirmatory factor analysis of the SCI.

Scale/Factor/Item	Factor loading	CR
**Self-care maintenance**		
Factor 1: Health-promoting behaviors		.73
Item 1	.58	
Item 3	.62	
Item 4	.67	
Item 7	.54	
Item 8	.54	
Factor 2: Illness-related behaviors		.66
Item 2	.67	
Item 5	.56	
Item 6	.65	
**Self-care monitoring**		.78
Item 9	.71	
Item 10	.70	
Item 11	.79	
Item 12	.73	
Item 13	.82	
**Self-care management**		
Factor 1: Autonomous behaviors		.77
Item 15	.70	
Item 16	.68	
Item 17	.68	
Item 20	.68	
Factor 2: Consulting behaviors		.87
Item 18	.87	
Item 19	.71	

SCI: Self-care Inventory; CR: composite reliability.

### Measurement invariance

As previously mentioned, the authors intended to recruit a larger than required sample size to perform measurement invariance across sex of participants. Three types of measurement invariance were examined in this study: equal form, equality of factor loadings, and equality of indicator intercepts. The equal form measurement invariance was supported for all three scales of the SCI. This was evidenced by the values of the goodness of model fit indices which met the criteria set a priori (RMSEA < .07, CFI > .95, and X^2^/df < 5). Regarding the equality of factor loadings invariance, the self-care monitoring and management scales demonstrated invariance as evidenced by the non-significant Chi-square statistic (see Table 4). For the equality of intercepts, measurement invariance was not supported for any of the SCI scales.

**Table 4:** Measurement invariance.

	Scale		
Invariance	Self-care Maintenance	Self-care Monitoring	Self-care Management
Equal form: RMSEA CFI X^2^/df	.045 .963 2.06	.037 .994 1.69	.049 .981 2.24
Equal factor loadings	X^2^ = 21.60, df = 8, p = .006	X^2^ = 7.03, df = 5, p = .22	X^2^ = 8.99, df = 6, p = .17
Equal intercepts	X^2^ = 110.84, df = 16, p < .001	X^2^ = 56.73, df = 10, p < .001	X^2^ = 38.16, df = 12, p < .001

RMSEA: root mean square error of approximation; CFI: comparative fit index; X^2^/df: chi-square/degrees of freedom.

### Internal consistency and bivariate correlations

The values of Cronbach’s α for all instruments used in the current study were acceptable (≥.70). In addition, the values of Cronbach’s α for three scales of SCI supported the internal consistency (See Table 5). Regarding Pearson’s r coefficients, the three scales of SCI and the SCSE demonstrated positive correlations that ranged between .56 and .70 (*p* < .01). Self-care maintenance scale was the only one with significant correlation with the CESD-R (*r* = -.10, *p* < .05). The bivariate correlation between the SCSE and CESD-R was also significant as presented in Table 5.

**Table 5:** Cronbach’s alpha and bivariate correlations.

	SCMain	SCMon	SCMang	SCSE	CESDR
SCMain	**.82**				
SCMon	.65**	**.86**			
SCMang	.60**	.70**	**.83**		
SCSE	.56**	.68**	.66**	**.93**	
CESDR	-.10*	.02	-.03	-.16**	**.95**

SCMain: self-care maintenance; SCMon: self-care monitoring; SCMang: self-care management; SCSE: self-care self-efficacy; CESDR: the Center for Epidemiologic Studies Depression Scale-revised.

**Correlation is significant at the 0.01 level (2-tailed).

* Correlation is significant at the 0.05 level (2-tailed).

Note: Values in bold (diagonal) represent the Cronbach’s α’s. Off-diagonals are the Pearson’s *r* correlations.

## Discussion

Self-care plays a key role in determining the health and well-being of healthy and ill people. There has been a growing interest among researchers to thoroughly understand how self-care affects various aspects of health among different populations. However, one of the main limitations of self-care research is the shortage of self-care measurement instruments that have empirical and theoretical support regarding psychometric properties [10, 11]. More specifically, there is a lack of research on the development and validation of comprehensive, theory-based self-care tools in the Arabic language and culture. We believed that the SCI [12], a new 20 items instrument to measure self-care in the general adult population, could be of use in the Arabic population. Therefore, the authors conducted this research to examine the psychometric properties of the Arabic version of the SCI in the general adult population in Jordan.

Before discussing the findings regarding the psychometric properties of the Arabic SCI, it is important to note that the mean scores of the three scales were lower than the cutoff point indicative of adequate self-care of persons with chronic illnesses measured with similar instruments (≥ 70) [27, 28]. In addition, the results of this study showed that the scores of self-care maintenance and self-care monitoring, measured with the SCI, are lower than the scores reported in a study conducted among the US adult population [12]. The scores of self-care management were comparable between this study and the study conducted by Luciani and colleagues [12], though. The differences in self-care maintenance and self-care monitoring could be attributed to cultural differences between the sample of the present study and previously conducted studies. From a theory-based standpoint, Riegel et al. [15] maintained that culture is a significant determinant of self-care in the general adult population. On the other hand, empirical evidence regarding such cultural variations in the general adult population is lacking. Previous research among persons with specific health conditions (e.g., heart failure) showed that cultural differences affect symptom recognition and health care practices [13, 14, 29]. Cultural differences could also affect the choice of adopting a curing not preventive healthcare attitude [30]. This evidence could explain the differences in the self-care maintenance and self-care monitoring between the current study and the findings reported by Luciani and colleagues [12]. However, it is important to note that the literature regarding cultural differences in self-care behaviors in the general adult population is still limited. Therefore, more research about how cultural values and beliefs affect self-care in the general adult population is necessary.

The CFA results in this study provide evidence about the factor structure of the three scales of SCI. This was evidenced by the goodness of model fit indices and factor loadings for the separate models of SCI and the simultaneous CFA. These results are consistent with the results of the first study about the psychometric properties of the SCI [12]. The number of factors under each SCI scale were the same in both studies; self-care monitoring (two factors), self-care monitoring (one factor), and self-care management (two factors). However, a minor difference between both studies should be highlighted. Including all items of self-care monitoring in the analysis of the current study showed that the model sufficiently fits the data. In contrast, one item (#8 How often do you avoid tobacco smoke) had to be removed in the study by Luciani and colleagues to improve the goodness of model fit indices [12].

The results of the simultaneous CFA in the present study showed that the three scales of the SCI adequately represent and reflect the main concepts (i.e., self-care monitoring, self-care maintenance, self-care management). The goodness of model fit indices and the factor loadings of the simultaneous CFA are in line with the results of the English version of the SCI [12]. As noted earlier, the SCI was developed based on the principles of the Middle range Theory of Chronic Illness and the Self-care of Chronic Illness Inventory [1, 15]. During the development of the SCI, some of the items pertinent to self-care of persons with chronic illness were modified to make the SCI applicable to the general adult population [12]. Meanwhile, the SCI encompasses the main concepts of the Middle range theory; namely, self-care maintenance, self-care monitoring, and self-care management. Collectively, the results reported here and by Luciani and colleagues [12] support the notion that SCI is a valid and reliable, theoretically grounded measure of self-care in the general adult population.

The results of this study showed that SCI has partial measurement invariance across participants’ sex. The equal form was the only type of measurement invariance that was exhibited by the three SCI scales. Such evidence illustrates that using the SCI to measure self-care in both male and female adults could provide valid, reliable measurement. However, exhibiting partial measurement invariance suggests that further research is warranted to validate the results of the current study. In addition, it is recommended that other factors that affect self-care should be investigated in the future (e.g., being well or having a chronic illness). Of note, measurement invariance analyses were not performed during the examination of the English version of the SCI, consequently, limiting the authors’ ability to discuss this topic with the context of previous research.

The values of the Cronbach’s α and composite reliability revealed that the internal consistency of the three scales of the SCI is supported. The values reported in this study are fairly comparable to the previously reported values [12]. An exception to this comparison was the composite reliability of the factor illness-related behaviors which is lower than the value reported by Luciani and colleagues [12]. While the present study findings indicate that the internal consistency of the SCI is supported, the researchers maintain that it is necessary to conduct test-retest analysis in the future to provide additional support for the reliability of the measure in the Arabic population.

The bivariate correlations showed that scores on the SCI scales are positively correlated with the level of self-care self-efficacy. All correlations were moderate and statistically significant. This is consistent with the results of the findings reported in the study regarding the validation of the original SCI [12]. Regarding the correlations with the scores on the CESD-R, the authors postulated that the SCI scales would exhibit negative correlations with participants’ depressive symptoms. However, there was only one statistically significant, weak correlation between CESD-R scores and self-care maintenance scores. This is inconsistent with previous research [16–18]. Future research is needed to address the inconsistency of the findings of the current study with previous research. One possible explanation for the absence of relationship between depressive symptoms and two scales of the SCI could be that depressive symptoms are typically viewed as a consequence of health conditions or illnesses (such as heart failure diabetes mellitus) and rather than a predictor of one’s self-care behaviors [31].

### Implications

There is a need for culturally relevant self-care instrument that can be used by Arabic-speaking adults in Jordan and the region. The authors intended to advance research regarding comprehensive measurement of self-care in Arabic-speaking adults. In addition, conducting this study was expected to facilitate examining the relationship between self-care and other variables such as physical health and psychological well-being. The results of this study showed that the factor structure, reliability, and measurement invariance of the Arabic version of the SCI are all supported. As a result, the authors recommend using the SCI as a comprehensive, theory-based measure of self-care in the general adult population. Using the SCI could, in turn, help identify the factors that influence adults’ self-care. It could also enable designing and implementing interventions directed toward improving self-care of the general adult population. The SCI could be used by healthcare providers and researchers (e.g., community and public health nurses) interested in self-care and health promotion in the general adult population.

### Limitations

The findings reported in this study are limited by the cross-sectional design used. Using convenience sampling is another main limitation of the present study. These limitations affect the generalizability of the results. In addition, the Arabic-speaking population is diverse and recruiting participants from Jordan only might limit the generalizability of the results to other countries and other population with different sociodemographic backgrounds. Therefore, future research with multiple data collection points is warranted. In addition, the authors recommend investigating the psychometric properties of the SCI among other Arabic-speaking population from different countries.

## Conclusion

The psychometric properties of the Arabic version of the SCI demonstrate its validity and reliability as a robust assessment tool for measuring self-care in the general adult population. This theory-based tool could enable researchers to achieve a deeper understanding of an individual’s self-care behaviors and their impact on the overall well-being. The authors recommend conducting further research to validate the results of the present study in other Arabic-speaking populations.
